# Invasive and Non-Invasive Congeners Show Similar Trait Shifts between Their Same Native and Non-Native Ranges

**DOI:** 10.1371/journal.pone.0082281

**Published:** 2013-12-17

**Authors:** Yedra García, Ragan M. Callaway, Alecu Diaconu, Daniel Montesinos

**Affiliations:** 1 Centre for Functional Ecology, Universidade de Coimbra, Coimbra, Portugal; 2 Division of Biological Sciences, University of Montana, Missoula, Montana, United States of America; 3 Biological Control Laboratory, Institute of Biological Research Iaşi, Iaşi, Romania; 4 Centro de Investigaciones sobre desertificación (CIDE CSIC-UV-GV), Montcada, València, Spain; Texas A&M University, United States of America

## Abstract

Differences in morphological or ecological traits expressed by exotic species between their native and non-native ranges are often interpreted as evidence for adaptation to new conditions in the non-native ranges. In turn this adaptation is often hypothesized to contribute to the successful invasion of these species. There is good evidence for rapid evolution by many exotic invasives, but the extent to which these evolutionary changes actually drive invasiveness is unclear. One approach to resolving the relationship between adaptive responses and successful invasion is to compare traits between populations from the native and non-native ranges for both exotic invaders and congeners that are exotic but not invasive. We compared a suite of morphological traits that are commonly tested in the literature in the context of invasion for three very closely related species of *Centaurea*, all of which are sympatric in the same native and non-native ranges in Europe and North America. Of these, *C. solstitialis* is highly invasive whereas C. *calcitrapa* and *C. sulphurea* are not. For all three species, plants from non-native populations showed similar shifts in key traits that have been identified in other studies as important putative adaptive responses to post-introduction invasion. For example, for all three species plants from populations in non-native ranges were (i) larger and (ii) produced seeds that germinated at higher rates. In fact, the non-invasive *C. calcitrapa* showed the strongest trait shift between ranges. *Centaurea solstitialis* was the only species for which plants from the non-native range increased allocation to defensive spines, and allocated proportionally less resources to reproduction, patterns contrary to what would be predicted by theory and other empirical studies to enhance invasion. Our results suggest caution when interpreting the commonly observed increase in size and reproductive capacity as factors that cause exotics to become invaders.

## Introduction

Many exotic organisms become much more abundant and have greater impact on other species in their non-native than in their native ranges [1], [2]. Many of these invasive species have been shown to evolve different trait expression in their non-native ranges [3]–[5]. These changes can be due to adaptation, genetic drift, hybridization and/or founder effects [6] and have the potential to contribute substantially to invasion. However, whether these changes cause invasive success is speculative. An opportunity to explore the causal link between morphological changes and invasion is to compare shifts expressed by exotic invasive species to those of exotic congeners that naturalize in their new habitats without becoming unusually abundant or having strong impacts [7], [8].

Comparing the traits of exotic species that differ in their ability to invade may help to understand the mechanisms that promote invasion [9]–[11]; but there have been fewer studies focusing on differences among exotic invasive and exotic non-invasive species [12]–[18]. Thus the combination of both approaches: 1) the study of exotic invasive and exotic non-invasive species, and 2) studying them in both their native and non-native ranges, has a great deal of potential to shed light on traits that might be important for invasive success and, perhaps more importantly, which adaptive trait shifts between native and non-native ranges may contribute the most to an exotic species evolving in a way to become more invasive [19].

A substantial body of literature shows a strong and general tendency for plants from populations in their non-native ranges to increase in size, germination rate, and reproductive output when compared with their native ranges [4]–[6], [20]–[25]. In turn, a less common response is the loss of herbivore defensive capacity by plants in non-native ranges [26]–[28], but when this occurs it is interpreted in the context of tradeoffs (the hypothesis of evolution of increased competitive ability, EICA) [20]. These evolutionary tradeoffs provide a major hypothesis for how exotic species might transform into invaders; however, to our knowledge there have been only a few works comparing inter-regional trait shifts among invasive and non-invasive congeners [16], [29].

We compared trait shifts for a set of three very closely related species: *Centaurea solstitialis*, *C. calcitrapa*, and *C. sulphurea*. All three species have highly overlapping ranges both within their native range of Spain and within their non-native range of California. To explore trait changes for these three congeners, we grew plants in common greenhouse conditions and asked the following questions: 1) is the invasive *C. solstitialis* inherently larger, more fecund, or better defended than the two non-invasive congeners, 2) have important trait shifts between the native and non-native ranges occurred only for the invasive *C. solstitialis*?

## Materials and Methods

*Centaurea solstitialis*, *C. calcitrapa*, and *C. sulphurea*, are closely related within the *Jacea* group of the *Centaurea* phylogeny [30]. These three species have overlapping distributions in their native ranges in Spain and also in their non-native ranges in California. *Centaurea solstitialis* has been introduced into California since at least 1824 [31]. Although Southern Europe is generally accepted as *C. solstitialis* native region, Prodan [as cited in 31] discussed that East Mediterranean and Caucasus are in fact the original source, while Western Mediterranean areas (like Spain) could have been colonized later. *C. calcitrapa's* introduction in California is thought to be about 1896 [32], [33] and for *C. sulphurea* at least 1923 [15], [34]. While *C. solstitialis* is likely to have experienced multiple introductions into California [35]–[37] and recent genetic analyses [38] show that, when compared with other regions in the native range (Hungary, Romania, Turkey and Georgia), Spanish populations have a genetic structure which is most similar to that of California and Argentina, thus confirming previous papers pointing to the importance of Spanish origin introductions on Californian plants [39], [40]. All populations studied occurred in or nearby roadsides in highly disturbed areas subjected to Mediterranean climate. Two of these neo-allopatric species, *C. solstitialis* and *C. calcitrap*a, occur over broad native (Southern Europe) and nonnative ranges (Americas, Australia), whereas *C. sulphurea* has a highly restricted native range in Spain and Morocco, and occurs as only a few populations in California [14], [29]. *Centaurea solstitialis* is much less common in Spain than *C. calcitrapa* [30] but *C. solstitialis* has become an aggressive invader in California, while *C. calcitrapa* has not. Based on herbarium records and our observations, *Centaurea sulphurea* is not common in either range (www.gbif.org). All three species inhabit the same ruderal habitats, are winter annuals (although *C. calcitrapa* can occasionally be bi-annual), form basal rosettes, and develop single bolting flower stems from the rosette. All three species also form large spines on their capitula, providing a common trait for which to compare allocation to defense.

In the summer of 2009, we collected seeds from fifteen different individuals from each of 45 different populations across the distributional range of the three species in Spain and California, USA (hereafter, “regions”; see Table S1). We sampled eight *C. solstitialis* populations from Spain and 11 populations from California, 10 *C. calcitrapa* populations from Spain and nine from California, and four *C. sulphurea* populations from Spain and three from California. No permissions were required for seed collection. The study species are considered weeds both in the native and non-native ranges and seed sampling was done on roadsides. We confirm that the field studies did not involve endangered or protected species. In January 2010, three seeds, randomly selected from each individual mother plant regardless of their achene type (pappus/non-pappus), were sown in each 2.2 L pot in a 50∶50 mix of 20–30 grit sand and local soil from natural grasslands near Missoula, Montana (total N = 675 plants). After germination, and before seedlings could experience any competitive effect on each other, exceeding seedlings were manually removed so that only one plant remained in each pot. Germination rates were sufficient to reach the intended number of replicates. All plants survived until the end of the experiment. A small proportion (3%) of *C. calcitrapa* plants did not reach reproductive state (it is occasionally biennial); such plants were evenly distributed among experimental groups; data from these plants was not used for the analyses. Plants were randomly mixed in a common garden greenhouse experiment with a temperature range of 10–35°C, watered every 1–2 days, and fertilized biweekly with 100 mL of 1.16 g L^−1^ Scotts Miracle-Gro (15∶30∶15 + micronutrients). Plants were grown until they flowered in a pollinator-excluded greenhouse, and we measured several variables during the germination and growth period: germination rate, rosette diameter, number of capitula, and spine length. Rosette diameter was measured, in mm, 30 and 90 days after sowing in order to calculate relative growth rates. We also manually cross pollinated plants by rubbing receptive capitula from two different individuals from the same natural population with each other. Since *C. solstitialis* is self-incompatible [29], crosses were randomly made between individuals within populations in order to obtain a seed-set from one flower per individual. Plants were harvested after capitula maturation (July, 2010), which was similar for all three study species. Harvested plants were dried for 48h at 70°C and weighed.

Data were analyzed with the statistical software R 2.15.2 [41] by means of linear mixed-effects models after Laird and Ware [42] but allowing for nested random effects of population. Species and regions (Spain or California) were fixed factors for each individual test, and the interaction between the two fixed factors was also studied. Tukey post-hoc tests were used by using R package “multcomp” and accounting with population as a nested random factor. Variables were transformed for normality when necessary.

## Results

**Figure 1:**
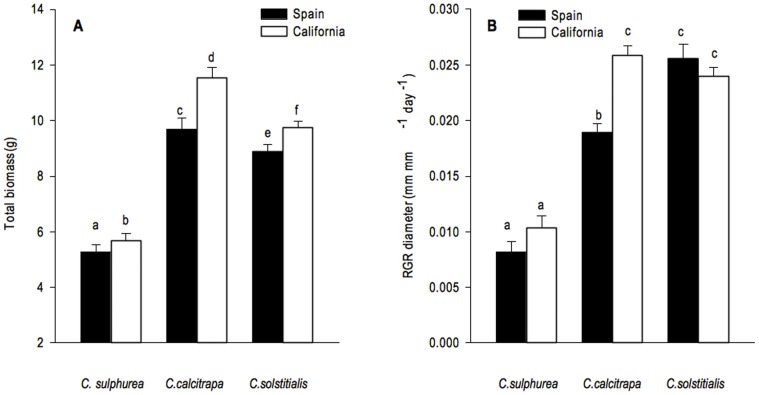
Total plant biomass (g; mean ± SE) (A); relative growth rate of rosette diameter (mm mm^−1^ day^−1^; mean ± SE) (B) for each species from each region. Different letters indicate statistically significant differences (*P*≤0.05).

For all populations and ranges combined, individuals of the non-invasive *C. calcitrapa* produced greater total mass than either of the other two species, *C. sosltitialis* was intermediate in total mass, and *C. sulphurea* was the smallest (Fig. 1A; *F_species_* = 15.46; df = 2,41 *P*<0.001; Tables S2, S3). The rosette relative growth rates (RGR) of *C. solstitialis* and *C. calcitrapa* were greater than those of *C. sulphurea* (Fig. 1B; *F_species_* = 43.32; df = 2,39 *P*<0.001).

**Figure 2:**
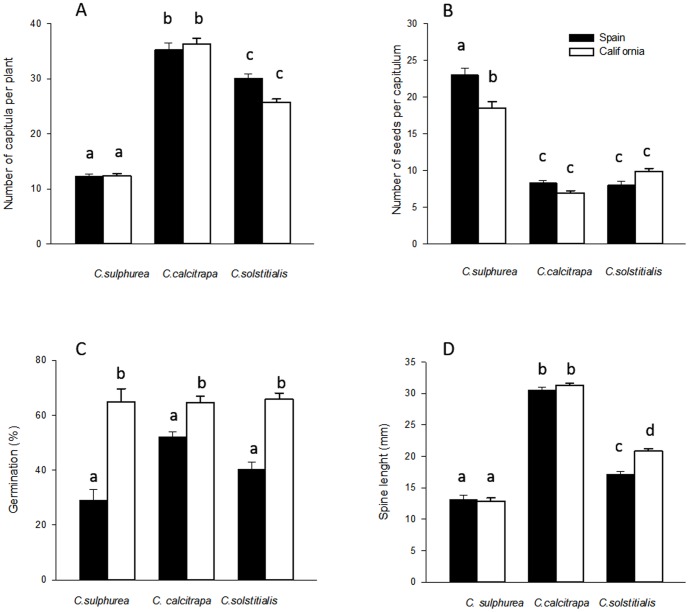
Number of capitula per plant (mean ± SE) (A); Number of seeds per capitulum (mean ± SE) (B); Germination percentage (mean ± SE) (C); Spine length (mm; mean ± SE) (D) for each species and region. Different letters indicate statistically significant differences (*P*≤0.05).

The non-invasive *C. calcitrapa* produced the highest number of capitula per plant, followed by *C. solstitialis* and then by *C. sulphurea* (Fig. 2A; *F_species_* = 43.84; df = 2,42; *P<*0.001; Tukey's post hoc *P<*0.001 for all species pairs). The number of seeds per capitulum was higher for *C. sulphurea* while *C.calcitrapa* and *C. solstitialis* did not differ (Fig. 2B; *F_species_* = 78.14; df = 2,3212; *P<*0.001; Table 1). There were no significant differences among species for seed germination rates (Fig. 2C; *F*_species_ = 1.85; df = 2,45; *P* = 0.168). *Centaurea solstitialis* seed-set is lower than previously reported [43], which could be due to natural variability within the species, or to a lower pollination efficiency of our manual treatment than that of natural insect pollinators. In any case, and since our tests compare data obtained from identical treatments, our results are informative at the comparative level.

**Table 1:** Percent increase in the mean trait value for plants from the non-native range, compared to mean of the trait from the native range.

Trait	*C. sulphurea*	*C. calcitrapa*	*C. solstitialis*
Biomass	**8%^a^**	**19%^b^**	**10%**
Rosette RGR	26%^a^	**36%^bc^**	−6%^c^
Capitula per plant	1%^a^	3%^b^	−15%^c^
Seeds per capitula	−20%^a^	−16% ^b^	22%^b^
Germination rate	**123%^a^**	**24%^a^**	**63%^a^**
Spine length	−2%^a^	2%^b^	**21% ^c^**

*P*≤0.05) and different letters indicate significant differences among species (*P*≤0.05). Numbers in bold indicate significance levels for inter-regional differences (

Across all populations and ranges *C. calcitrapa* had longer spines than either of the two congeners, *C*. sosltitialis was intermediate, and *C. sulphurea* produced the smallest spines (Fig. 2D; *F*_species_ = 278.07; df = 2,39; *P*<0.001; Tukey's post hoc *P<*0.001 for all species pairs).

For all three species, individual plants grown from seed collected in the non-native ranges produced more total biomass than plants grown from seed collected in the native region (*F_region_* = 4.07; df = 1,41; *P* = 0.051), but the largest shift in this trait was expressed by the non-invasive *C. calcitrapa* (Fig. 1A, Table 1). *Centaurea calcitrapa* was the only species to show an inter-regional shift in rosette RGR, with plants from California growing faster than plants from Spain (Fig. 1B, Table 1; *F_region_* = 3.91; df = 1,39; *P* = 0.055, *F_species x region_* = 3.79; df = 2,39; *P* = 0.031; Tukey's post hoc *P* = 0.007). We found no differences in the number of capitula per plant between native and non-native regions for any of the three species (Fig. 2A; *F_region_* = 0.49; df = 1,41; *P* = 0.487). *Centaurea sulphurea* was the only species that showed a shift in the number of seeds per capitulum between regions, with Californian plants producing fewer seeds per capitulum than Spanish plants (Fig. 2B, Table 1; *F_region_* = 78.13; df = 2,3212; *P* = 0.005; *F_species x region_* = 0.057). Seeds produced by plants of all species from the non-native range had higher germination rates, with the average increase across the three species of 61% when compared to the native region (Fig. 2C, Table 1; *F_region_* = 22.90; df = 1,45; *P*<0.001). *Centaurea sulphurea* experienced the highest change in germination rate between ranges followed by *C. solstitialis* (*P* = 0.006; *P* = 0.002). *Centaurea solstitialis* plants from California were the only species that demonstrated an increase in spine length in the non-native range, with Californian plants producing spines that were 21% longer than their conspecifics from the native range (Fig. 2D; *F_region_* = 8.86; df = 1,39; *P = *0.005; *F_species x region_* = 4.39; df = 2,39; *P* = 0.019).

## Discussion

In general, traits related to growth, fecundity, and defense that we measured were not inherently greater in value for the exotic invasive *C. solstitialis* than for non-invasive congeners. More importantly, the length of spines on the calyces was the only trait for which *C. solstitialis* demonstrated a greater increase in the non-native range than the other congeners. Thus the most important interpretation of our results is that, for these species and traits, we found no evidence that the invasive species exhibited any stronger shifts in key traits than very similar but non-invasive exotic congeners.

Our results are consistent with a large body of literature demonstrating substantial differences in morphology or size for plants from populations in the native and non-native ranges. For example, an increase in size for plants in the non-native ranges has been widely documented and overall, invasive species tend to have higher values also for leaf-area allocation, shoot allocation, growth rate, physiology, and fitness [5], [6], [19], [20], [22], [23], [43]–[45]. Seed and seedling size has been demonstrated to differ between native and non-native ranges of *C. solstitialis*, but not for the non-invasive *C. calcitrapa* or *C. sulphurea* [16]. However seedling size only differed when *C. solstitialis* was grown in competition with the European native grass *Bromus hordeaceus*, when Californian individuals grew more than their Spanish counterparts, but not when in competition with the American native *Poa secunda*, thus showing that individuals from the non-native range present increased competitive ability under certain circumstances [16]. Germination rates have been tested much less, but Hierro *et al.* [40] found higher germination rates for seeds produced on *C. solstitialis* plants from the non-native range of California than plants from the native range of Turkey in a common garden; Ridenour *et al.* [5] found that the mean germination rate of *C. stoebe* from non-native North American populations was 81% higher than that of native European populations. Kudoh *et al.* [46] observed patterns consistent with adaptation to fall germination for invasive strains of *Cardamine hirsuta*. These results indicate that exotic plants may experience strong selective pressure in their non-native ranges and respond to this pressure rapidly; a rapid accumulation of ecological adaptations which has been found to lead to incipient degrees of reproductive isolation between native and non-native ranges of introduced species [29]. However, our results for these *Centaurea* congeners suggest caution in interpreting these apparent adaptive responses as *drivers* of invasion. Both exotic invasive and non-invasive congeners exhibited substantial trait shifts, potentially very important for local adaptation but, in view of our results, not necessarily key to the dramatic biogeographic shifts in abundance and impact manifest by *C. solstitialis* but not *C. calcitrapa* or *C. sulphurea*.

Invasive species often grow much faster than the native species they exclude [16], [18], [47]–[49]. Graebner *et al*. [16] found that, when in competition with native grass species, the relative growth rate (RGR) of *C. solstitialis* biomass, from seed to seedling, was greater than that of *C. calcitrapa* or *C. sulphurea*, but there was no difference in this trait between the native and non-native ranges for any of the three congeners. Here, we also found that the overall RGR of *C. solstitialis* rosettes was 11% greater than that of *C. calcitrapa* and 42% greater than that of *C. sulphurea*, but that only *C. calcitrapa* showed evidence for evolving higher RGR in the non-native range. Perhaps these inherently rapid growth rates were critical for the early success of *C. solstitialis* relative to its congeners.

*Centaurea solstitialis* was intermediate in spine length, but the only species to produce significantly larger spines in California than in Spain. Increased spine length could simply be correlated with an increase in total biomass, but spine length augmented 21% in non-native populations; whereas biomass increased 10%. Given that *C. solstitialis* in California often occurs with domestic livestock, perhaps this strong generalist herbivore pressure is a more important selective force in the non-native range of California. Indeed, cows, sheep and goats have been described as effective grazers of *C. solstitialis* and have been effectively used as a control agent in California [50], [51].

Time since introduction is an important variable when studying biological invasions, and time lags of more than 50 years between introduction and invasion are common [52], [53]. Several multispecies studies found a positive relationship between the capacity to spread of exotic plants and the time since their introduction in the non-native areas [54]–[56]. The cause of the lags is partly inherent to the dynamics of population growth and range expansion [57], [58] but frequently it is also related to the need to accumulate sufficient genetic diversity via repeated introductions; or to develop key adaptations to the non-native range which are crucial for invasive success [19], [52], [57]. This has generated a phenomena called “invasion debt”, a hypothesis suggesting that past human activities involving the introduction of exotic species will have a future impact on ecosystems after a time lag since date of introduction [59]–[61]. The three species considered in this study differ in time since introduction, from 189 years for the invasive *C. solstitialis*, to 117 years and 90 years for the non-invasive *C. calcitrapa* and *C. sulphurea*, respectively. Interestingly, our results show that these three closely related species show a similar amount of trait-shifts, suggesting that trait-shifts can happen very rapidly after introduction although, visibly, they do not necessarily lead to invasive success at the same speed.

A number of studies have attempted to elucidate the key traits that drive invasions [18], [62]–[64]. These efforts however, have led to the conclusion that the important traits may differ among species, and even among sites for the same invasive species. For instance, Hierro et al. [40] studied germination patterns for *C. sosltitialis*, and found differences not only between native and non-native ranges, but also between populations from the non-native ranges of Argentina and California. Previous studies [16] with the species system used in the present work found competitive advantages related to seed and seedling size of *C. solstitialis* under certain circumstances; however, both studies considered different variables under different conditions, and the absence of significance for some of the studied variables does not preclude the possibility of some key variable not being considered.

Multispecies comparisons have been fruitful, but tend to compare invasive species with natives within the invaded region [18], [65], [66]. The trait differences detected so far between native and non-native species could be due in part more to their different geographic origins, and the likelihood of being transport than to actually advantageous traits [66]. Traits have been compared among several naturalized invasive and non-invasive species. For instance Muth and Pigliucci [15] compared *Crepis* and *Centaurea* in their non-native range, and found that invasiveness corresponded well with species-specific trait interactions and introduction histories. This approach does not explain if the differentiating traits are acquired after introduction or were present in the native range, thus studies involving individuals from both the native and the non-native ranges are necessary [19]. Our results do not preclude that a unique and synergistic combination of local adaptations drive invasion success. However, since we found that non-invasive species show some of the same trait shifts between native and non-native regions as an invasive species, our results suggest caution in assuming that shifts in traits thought to be important to invasion actually cause the invasive success of a particular species.

## Supporting Information

Table S1 **Location of** ***Centaurea*** **populations for each of the species from each of the studied regions.** Latitude and longitude coordinates are datum WGS84.
(DOCX)

Table S2 **Statistical results for the Linear Mixed Models for each studied trait and factor.** Significant differences are in bold.
(DOCX)

Table S3 **Tukey's post-hoc p-values following Linear Mixed Models for species to species comparisons for each trait.** Significant differences are in bold, n/a: not applicable.
(DOCX)
