# Supplementary material for: Invasive and Non-Invasive Congeners Show Similar Trait Shifts between Their Same Native and Non-Native Ranges
Source: PLoS One. 2013 Dec 17;8(12):e82281. doi: 10.1371/journal.pone.0082281 (PMC3866105; doi:10.1371/journal.pone.0082281)
Supplement: Table S1 — Location of Centaurea populations for each of the species from each of the studied regions. Latitude and longitude coordinates are datum WGS84. (DOCX) [file pone.0082281.s001.docx]

**Table S1. Location of *Centaurea* populations for each of the species from each of the studied regions.** Latitude and longitude coordinates are datum WGS84.
