# Supplementary material for: Invasive and Non-Invasive Congeners Show Similar Trait Shifts between Their Same Native and Non-Native Ranges
Source: PLoS One. 2013 Dec 17;8(12):e82281. doi: 10.1371/journal.pone.0082281 (PMC3866105; doi:10.1371/journal.pone.0082281)
Supplement: Table S2 — Statistical results for the Linear Mixed Models for each studied trait and factor. Significant differences are in bold. (DOCX) [file pone.0082281.s002.docx]

**Table S2. Statistical results for the Linear Mixed Models for each studied trait and factor.**

| **Trait** | **Species** | **Region** | **Species x Region** |
| --- | --- | --- | --- |
| Biomass | ***P* < 0.001** | ***P* = 0.050** | *P* = 0.506 |
| Rosette RGR | ***P* < 0.001** | ***P* = 0.055** | ***P* = 0.031** |
| Capitula per plant | ***P* < 0.001** | *P* = 0.483 | *P* = 0.190 |
| Seeds per capitula | ***P* < 0.001** | ***P* = 0.005** | ***P* = 0.057** |
| Germination rate | *P* = 0.168 | ***P* < 0.001** | *P* = 0.161 |
| Spine length | ***P* < 0.001** | ***P* = 0.005** | ***P* = 0.019** |

Significant differences are in bold.
