# Supplementary material for: Invasive and Non-Invasive Congeners Show Similar Trait Shifts between Their Same Native and Non-Native Ranges
Source: PLoS One. 2013 Dec 17;8(12):e82281. doi: 10.1371/journal.pone.0082281 (PMC3866105; doi:10.1371/journal.pone.0082281)
Supplement: Table S3 — Tukey's post-hoc p-values following Linear Mixed Models for species to species comparisons for each trait. Significant differences are in bold, n/a: not applicable. (DOCX) [file pone.0082281.s003.docx]

**Table S3.** **Tukey’s post-hoc p-values following Linear Mixed Models for species to species comparisons for each trait.**

| **Trait** | ***C. solstitialis* vs. *C. calcitrapa*** | ***C. calcitrapa* vs. *C.sulphurea*** | ***C. sulphurea* vs. *C. solstitialis*** |
| --- | --- | --- | --- |
| Biomass | ***P* = 0.057** | ***P* < 0.001** | ***P* < 0.001** |
| Rosette RGR | *P* = 0.680 | ***P* < 0.001** | ***P* < 0.001** |
| Capitula per plant | ***P* < 0.001** | ***P* < 0.001** | ***P* < 0.001** |
| Seeds per capitula | *P* = 0.110 | ***P* < 0.001** | ***P* < 0.001** |
| Germination rate | n/a | n/a | n/a |
| Spine length | ***P* < 0.001** | ***P* < 0.001** | ***P* < 0.001** |

Significant differences are in bold, n/a: not applicable.
